# Winning hearts and minds: ECG reporting in the first seizure clinic

**DOI:** 10.1186/s12872-021-02174-4

**Published:** 2021-07-31

**Authors:** Xuya Huang, N. Malek, J. Simpson, D. Kalladka, F. G. Dunn, J. P. Leach

**Affiliations:** 1 Department of Neurology, Institute of Neurosciences, Queen Elizabeth University Hospital, Glasgow, G51 4TF UK; 2 Department of Neurology, Ipswich Hospital NHS Trust, Ipswich, UK; 3 British Heart Foundation, Institute of Cardiovascular and Medical Sciences, University of Glasgow, University Avenue, Glasgow, UK; 4 Directorate of Stroke and Neurosciences, Imperial College Healthcare NHS Trust, Charing Cross Hospital, Fulham Palace Road, London, UK; 5 School of Medicine, University of Glasgow, Glasgow, UK

**Keywords:** Epilepsy, Seizure, Syncope, ECG

## Abstract

**Background and aims:**

An electrocardiogram (ECG) is a mandatory test for anyone presenting with loss of consciousness. Many referrals to the first seizure clinic (FSC) are caused by syncope. We assessed the sensitivity of neurologists’ ECG reporting in detecting rhythm abnormalities including some potentially life-threatening cardiac conditions.

**Methods:**

We audited patients referred to a FSC in Glasgow over 4 years. All ECGs were interpreted by the attending neurologist as standard practice. Subsequently, two cardiologists reviewed the ECGs independently.

**Results:**

Of 160 consecutive patients, 92 patients (58%) were diagnosed as having seizures, 43 (27%) as syncope, and 25 (16%) were unclassified. Twenty eight ECGs thought to be normal by the neurologist were considered abnormal by the cardiologist, including three with long corrected QT interval. The proportion of abnormal ECGs and disparity in reporting between neurologists and cardiologists persisted independent of the underlying diagnosis.

**Conclusion:**

Reporting of ECGs by non-cardiologists may not be adequately sensitive in picking up potentially life threatening cardiac conditions. Cardiologist input into FSCs is recommended to enhance the diagnostic yield.

## Introduction

A first seizure clinic (FSC) is a rapid access clinic assessing patients who presented with an episode of loss of consciousness and is suspicious of seizure activity. Most patients have been assessed by general physicians, and had investigations such as metabolic screen, brain imaging and an electrocardiogram (ECG) prior to the referral to the FSC. Any definitively diagnosed cardiac syncope based on obvious or potentially serious arrhythmias such as ventricular tachycardia and second or third degree Atrioventricular (AV) block from the emergency department are directly referred to cardiologists for further investigations. Referrals to first seizure clinic arise for a variety of reasons, most commonly relating to epilepsy or syncope [1]. Syncope is the most common mimic of seizure [6] and will sometimes arise from cardiac pathologies which may be difficult to differentiate from epilepsy [1]. Twelve-lead ECG is a simple, non-invasive test which can identify cardiac pathology in patients with a previous history of convulsive episodes or a diagnosis of epilepsy [1]. Guidelines state that all patients presenting to a first seizure clinic should undergo 12 lead ECG recording [7]. In many general clinics ECG tracings (along with their automated reporting) may well be reviewed and interpreted by someone who is not trained in cardiology. The sensitivity and specificity of such non-specialist reporting is unknown, and it is possible that a lack of sensitivity may fail to identify underlying cardiac conditions. We audited the specificity and sensitivity of non-cardiologists’ reporting in patients having had a single episode of loss of consciousness.

## Methods

We collected 12-lead ECG recordings from patients attending a first seizure clinic in Glasgow. All patients were seen at least once by the same neurologist (JPL) over four years. All patients who were included in this study were outpatient referrals who were otherwise clinically well. The ones who were acutely unwell at the time of loss of consciousness (LoC) would have had an emergency admission and undergone necessary investigations to exclude acute pathology such as pulmonary embolism. Only those with LoC with no apparent secondary causes were referred to the first seizure clinic. Patients’ demographic details, the diagnostic conclusion reached by the neurologist at the initial visit and current health status were documented from electronic patient records using a standardised data collection tool. Patients with previous ECG recordings were excluded. All ECGs were then reviewed independently by two cardiologists blinded to patient details. The ECG reports between the neurologist and cardiologist were compared. The ECG was considered abnormal if there was a prolonged corrected QT (QTc) interval defined as QTc > 450 ms, short PR interval (PR interval ≤ 100 ms), left axis deviation, voltage criteria for left ventricular hypertrophy (LVH) by Sokolow-Lyon criteria, heart block of any degree, left or right bundle branch block defined as QRS duration ≥ 120 ms, any significant arrhythmia, bradycardia, tachycardia or ST-segment or T wave changes.

The final diagnosis reached by the neurologist (seizure, syncope, or not known) was collected from the case notes. Inter-observer agreement (Cohen’s Kappa) was calculated using Graphad Prism software version 6.0 for windows (La Jolla CA, USA).

## Results

In a FSC run by one neurologist in a tertiary care neurology referral centre in the West of Scotland between 2009 and 2012, 160 consecutive patients were identified who had no previous ECG recording available. The demographic details of the patients are presented in Table 1.

**Table 1:** Patient demographic details

	Seizure (n = 92)	Syncope (n = 43)	Unclassifiable^b^ (n = 25)
Age (y) median (IQR^a^)	37 (23–55)	35 (24–48)	32 (23–47)
Male (n, %)	54 (58.7%)	26 (60.5%)	12 (48%)
On QT prolonging medication (n, %)	4 (4.3%)	4 (9.3%)	5 (20%)
History of heart disease (n, %)	2 (2.2%)	4 (9.3%)	0 (0%)
Family history of epilepsy (n, %)	4 (4.3%)	2 (4.7%)	1 (4%)
Number of episodes pre-referral median (IQR)	1 (1–1)	1 (1–2)	1 (1–1)

^a^Interquartile range

^b^At initial presentation

92 (58%) cases were thought to have had epilepsy or seizure; 43 (27%) were thought to have an episode of syncope. Patients who were thought to have experienced syncope and required further cardiac investigations, were referred to cardiology regardless of their ECG interpretation. In 25 cases (16%) the cause for LoC was uncertain.

**Fig. 1:**
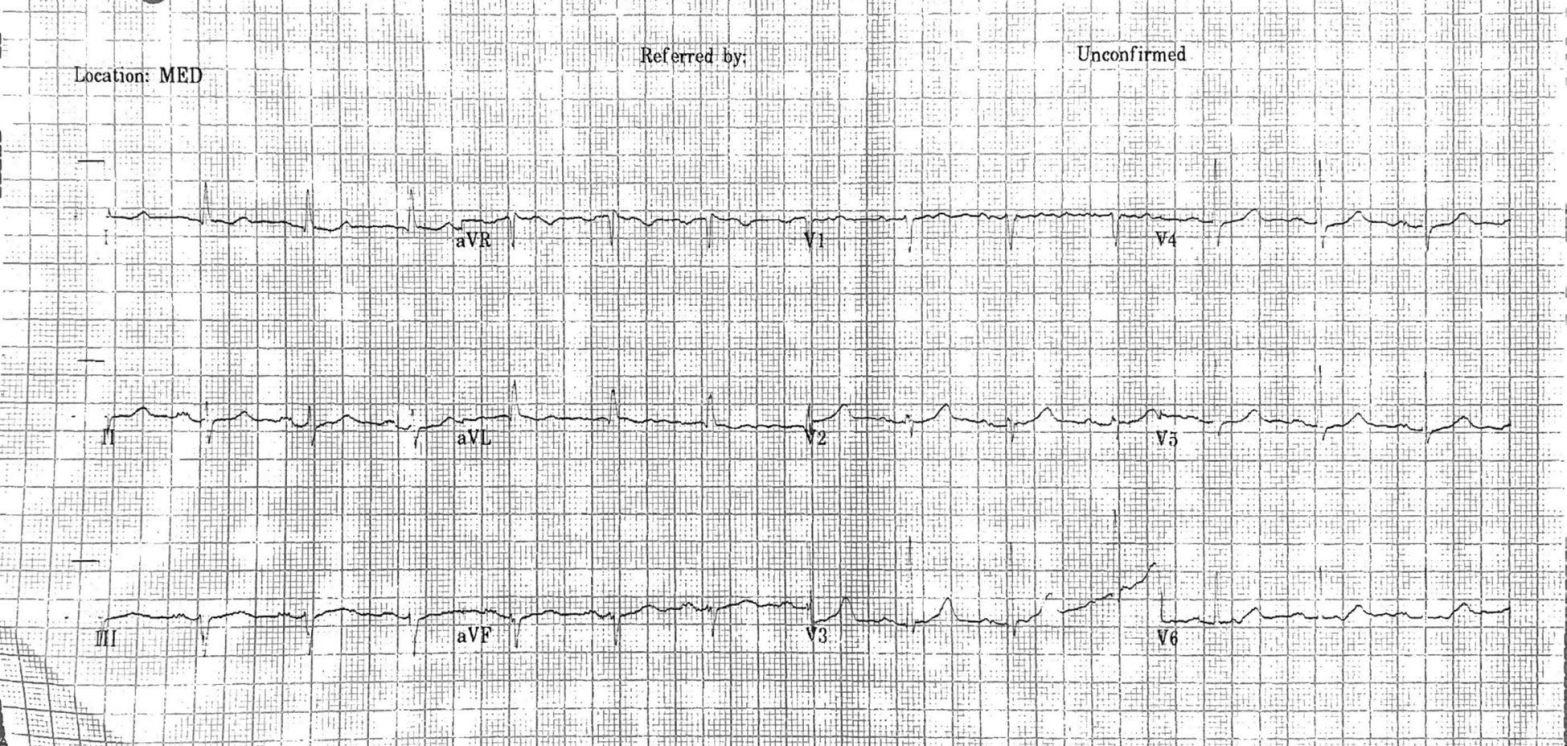
A sample ECG that was interpreted as normal by the neurologist, and subsequently thought to have a prolonged QTc by the reviewing cardiologists

The ECG reporting by the cardiologist and the neurologist was compared (Table 2). The neurologist reported 16 patients (10%) as having significant abnormalities on the initial ECG. In contrast, 39 (25%) were judged abnormal by the reporting cardiologists. A sample ECG that was thought to be normal by the neurologist, and deemed to have prolonged QTc by the review cardiologists is showed in Fig. 1.

**Table 2:** Comparison of significant ECG abnormalities by specialist

	Neurologist reading (n)	Cardiologist reading (n)	Cardiology referral (n)
Seizure (n = 92)			
Prolonged QT (> 450 ms)	6	8	4
Left axis deviation	2	0	0
Short PR interval (PR ≤ 100 ms)	1	3	0
ST-T wave abnormalities	0	5	0
Left ventricular hypertrophy	1	7	1
First degree heart block	0	0	0
Left bundle branch block	1	1	0
Right bundle brunch block	0	0	0
Syncope (n = 43)			
Prolonged QT (> 450 ms)	1	1	0
Left axis deviation	0	0	0
Short PR interval (PR ≤ 100 ms)	0	1	0
ST-T wave abnormalities	0	0	0
Left ventricular hypertrophy	0	4	0
First degree heart block	1	1	0
Left bundle branch block	0	0	0
Right bundle brunch block	0	0	0
Unclassifiable (n = 25)			
Prolonged QT (> 450 ms)	2	3	0
Left axis deviation	0	0	0
Short PR interval (PR ≤ 100 ms)	0	1	0
ST-T wave abnormalities	0	2	0
Left ventricular hypertrophy	0	0	0
First degree heart block	0	0	0
Left bundle branch block	0	0	0
Right bundle brunch block	2	2	0

The ECG reports were discordant in 37 cases (Table 3). Five patients had further input by a cardiologist at the time. Among 39 patients who had significant abnormalities on their 12-lead ECGs, three had a documented history of cardiac disease (all had history of ischemic heart disease, two with previous myocardial infarction, and one had left ventricle dysfunction).

**Table 3:** Comparison of ECG reporting (normal versus abnormal) by the reporting neurologist and the cardiologist

			Inter-observer agreement
	Normal By cardiologist	Abnormal By cardiologist	Kappa value (SE, CI)
Normal by neurologist	121	28	0.304 (0.09, 0.14–0.47)
Abnormal by neurologist	5	11	

*SE* standard error, *CI* confidence interval

The cardiologists identified three additional patients with prolonged corrected QT intervals. None of them had cardiology input after attendance at first seizure clinic. After a median time of 5 years follow up, there have been no deaths in the 28 patients whose ECG abnormalities were only picked up on review by the cardiologists at the time of study.

There were no changes made to the classification of cause of LoC after the ECG reading by the cardiologists (Table 4). However, during subsequent follow-up, one patient initially diagnosed with syncope was thought to have seizure, and three patients with initially unclassified episodes were subsequently thought to have seizures. Serious significant ECG abnormalities including prolonged corrected QT interval or heart block were detected in 13 patients (12 had prolonged QT interval, and 1 had first degree heart block). Of the twelve patients with prolonged QTc, five had input from cardiologist at the time either as new referrals or they were already known to cardiology. They have all been investigated further to identify the causes of the prolonged QTc including, but not limited to multidisplinary team discussion of the case, ambulatory cardiac monitoring, possibly genetic testing. Two (16.7%) were prescribed drugs known to prolong the QT interval (citalopram or amitriptyline); one initially thought to be congenital, with negative genetic testing. Another two were deemed to have metabolic causes, with QTc returning to normal following correction. Patients without ECG abnormality, did not undergo investigations to look for primary causes of the QTc prolongation. No other ECG abnormalities such as Brugada syndrome, bifascicular, trifascicular, or complete heart block were detected. Other cardiac investigations such as Holter monitoring or upright tilt-table test when deemed appropriate by the cardiologist in individual cases were ordered by them.

**Table 4:** Comparison of initial versus eventual diagnosis in each diagnostic group

	Seizure (n)	Syncope (n)	Unclassifiable (n)
Initial diagnosis	92	43	25
Eventual diagnosis	95	42	23

The incidence of ECG abnormalities was examined in each of the initial diagnostic groupings. Among those patients thought to have had a seizure, the neurologist determined that there were 11 abnormal ECGs, while the cardiologists reported 24 abnormalities, a difference of 15% between the two reporters. Of the cardiologists’ reports there were 8 recordings showing prolonged corrected QT intervals and 3 showing short PR intervals.

Among the 43 patients thought to have had syncope, there was more concordance between the cardiology and neurology opinion, with rates of abnormalities in neurologist and cardiologists reporting being 2 and 7 patients respectively (a difference of 8%), including only one short PR interval and no patients with a prolonged corrected QT interval.

In 25 patients with no clear initial diagnosis reached, the neurologist and cardiologists ascertained abnormality in 2 and 4 patients respectively (a difference of 8%), with one shortened PR interval and one prolonged QT interval picked up only by the cardiologists.

## Discussion

Misdiagnosis of epilepsy is a well-recognised problem [8]. This misdiagnosis rate can be as high as a quarter of all the cases seen in a specialist clinic [8]. Potential reasons for misdiagnosis of epilepsy include underlying cardiac disease, particularly cardiac rhythm disorders, masquerading as provoked or unprovoked ‘seizures’ [10]. Failure to correctly diagnose cardiac rhythm disorders in such patients leaves them exposed to inappropriate and sometimes dangerous anti-epileptic medications [4]. Furthermore, in such cases, where an underling cardiac condition is undiagnosed and left untreated, this places these patients at an unacceptable risk of potentially fatal cardiac arrhythmias [3]. For this reason, a 12 lead ECG is mandatory in every patient attending a first seizure clinic [7]. It remains a cheap, quick and non-invasive screening test which has good sensitivity and specificity in diagnosis and exclusion of serious cardiac disorders when utilised by someone with appropriate expertise. First seizure clinics are usually run by neurologists. Lack of cardiology input, even with automated computerised reporting, may leave some cardiac rhythm disorders undiagnosed.

In our cohort of 160 patients, around 60% had an initial diagnosis of epilepsy or first seizure while 27% were diagnosed as having experienced syncope. We accept that many of those with cardiac causes of LoC would have been picked up by the referring teams and referred directly to cardiology. While the initial diagnosis was changed in only a small minority of patients, there was a disparity in the results of the ECG interpretation between the neurologist and the cardiologist. The requesting neurologist uncovered 10 cases of abnormal QT or PR interval, with a further 7 only picked up by the cardiologist. Importantly, among 149 patients thought to have a normal ECG by the neurologist, 28 (19%) had an abnormality uncovered on re-analysis by the cardiologist. Fortunately, in this small cohort, none of the patients with a missed ECG abnormality came to harm, but we feel it is important to reduce this possibility.

While previous studies have reported the incidence of abnormal ECGs in patients attending a first seizure clinic [9], to our knowledge, this is the only study has examined the differential rates of ECG abnormality reporting between specialist and non-specialist clinicians, therefore highlighting the need of cardiologist specialist input in first seizure clinic.

We would argue that in the interest of patient safety, even where modern ECG machines have built-in automated reporting software, it is beneficial to have cardiology reporting of ECGs carried out in first seizure clinics. This would not only enhance the accuracy of the interpretation of the ECGs, but will also identify patients that require cardiology review and investigation. This work would support the practice in secondary and tertiary referral centres of providing rapid access to a ‘black-out’ clinic [2, 5] with specialist input from cardiology, a neurology, geriatrician and—where available—a clinical autonomic physiologist. At the least, we have demonstrated that a significant benefit of second reporting of the ECG by cardiologists will enhance patient safety and subsequent targeted rapid cardiology review. In our clinical practice, work is ongoing to encourage cooperation between cardiologists and neurologist in this clinical setting. We feel it is therefore important to disseminate our findings to raise awareness and promote change to improve patient safety.

This study is limited in scope as it is a retrospective cohort study from a single neurologist’s first seizure clinic. However, it has demonstrated a potential for improvement of patient safety. It could be validated in a larger cohort recruiting patients from multiple centres.

## Conclusions

A twelve lead ECG is a useful test in a first seizure clinic to rule out cardiac rhythm abnormalities, but relying on a neurologist to detect significant cardiac problem in a FSC is inadequately sensitive, as demonstrated from our audit. We would recommend that ECGs from a FSC should be interpreted by a trained cardiologist.

## Data Availability

Data is available from the corresponding author by request.

## Abbreviations

FSC: First seizure clinic
ECG: Electrocardiogram
AV: Atrioventricular
LoC: Loss of consciousness
QTc: Corrected QT
LVH: Left ventricular hypertrophy
